# Characterization upon electrical hysteresis and thermal diffusion of TiAl_3_O_*x *_dielectric film

**DOI:** 10.1186/1556-276X-6-557

**Published:** 2011-10-19

**Authors:** Lei Shi, Zhiguo Liu

**Affiliations:** 1 Hefei National Laboratory for Physical Sciences at the Microscale, University of Science and Technology of China, Hefei, Anhui 230026, PR China; 2 National Laboratory of Solid State Microstructures, Nanjing University, Hankou Road 22, Nanjing, 210093, People's Republic of PR China

**Keywords:** electrical hysteresis, thermal diffusion, pulsed laser deposition

## Abstract

In this paper, we have investigated the electrical properties of TiAl_3_O_*x *_film as electrical gate insulator deposited by pulsed laser deposition and presented a simple method to describe the thermal diffusion behaviors of metal atoms at TiAl_3_O_*x*_/Si interfacial region in detail. The TiAl_3_O_*x *_films show obvious electrical hysteresis by the capacitance-voltage measurements after post-annealing treatment. By virtue of the diffusion models composed of TiAl_3_O_*x *_film and silicon, the diffusion coefficient and the diffusion activation energy of the Ti and Al atoms are extracted. It is valuable to further investigate the pseudobinary oxide system in practice.

**PACS: **77.55.-g; 81.15.Fg; 81.40.Gh.

## Introduction

High electrical permittivity (*k*) insulators are presently investigated as possible replacements of SiO_2 _or SiON film gate dielectric in order to hurdle the increased tunneling leakage current while the devices are further scaled down. Although a huge number of single-phase high-*k *materials have been investigated, there is no suitable material that could completely replace the traditional SiO_2 _as gate dielectrics [1-4]. It is exciting that the recent researches have been concentrated upon the pseudobinary oxides mostly. This section focuses on all these candidates in an attempt to combine and complement the desirable properties from different materials, and then overcome the deficiencies associated with the individual material. Furthermore, the criteria for possible replacement dielectrics require that those materials are chemically stable on silicon substrates at high temperature as well. In fact, most of these potential candidates might partially react with silicon substrates at the interface after thermal treatment, and thus cause an increase of leakage current and degrade devices performance with high-*k *gate dielectrics [5,6]. As for a potential candidate, it is very important to pursue a small interfacial reaction for a perfect gate dielectrical candidate, except the preferable electrical properties.

In our earlier research, the electrical properties have shown that the TiO_2_-incorporated Al_2_O_3 _film may be a potential replacement as gate dielectrics because of its high dielectric constant, low leakage current, low charge density, etc. [7]. Nevertheless, the interfacial reaction between the film and silicon substrate has been observed, despite of the improved device performance. The interfacial thickness, along with the leakage current, rises rapidly with the increase of the annealing temperature. This is very damaging for high-*k *gate material in electrical devices. In order to describe this behavior in detail, we have deposited the TiAl_3_O_*x *_films as much as micron of magnitude, and then, a longtime annealing treatment is adopted to drive all atoms in the film close to the balance state. Thus, the diffusion coupling model for the interfacial layer has been constructed, from which the atom diffusion properties may be extracted if the film is thick enough. In this paper, the TiAl_3_O_*x *_films have been deposited on silicon substrate successfully by pulsed laser deposition method, and characterized systematically by virtue of recording the electrical properties and the microstructure of the interfacial layer. With the diffusion coupling model of TiAl_3_O_*x *_film and silicon, the diffusion coefficient and the diffusion activation energy of the Ti and Al atoms are extracted. This is a part of effort to understand the TiAl_3_O_*x *_film in the future research.

## Materials and methods

The pseudobinary TiAl_3_O_*x *_thin films were deposited by a pulsed laser deposition procedure with different thickness for different measure propose. Before the deposition, the substrates were ultrasonically cleaned by acetone and de-ionized water in turn, and then immersed in the diluted hydrofluoric acid solution to remove the native silicon dioxide, thus leaving a hydrogen-terminated silicon surface. The TiAl_3_O_*x *_films were grown on the treated silicon substrate at 673 K under a low oxygen partial pressure of 6.0 × 10^-5 ^Pa. After the deposition process, some of the films were *in situ *annealed at 673 K for 20 min to reduce the defects in the films as possible. To characterize the electric properties of the TiAl_3_O_*x *_films with the capacitance-voltage (*C*-*V*) measurement, the corresponding films were deposited on the abovementioned available silicon substrates to form metal-oxide-semiconductor capacitor (MOS) structures with platinum electrode.

To further analyze the atom distribution at higher temperature, the films were post-annealed at 773, 873, 973, and 1,073 K for 6 h, respectively. High-resolution transmission electron microscopy (HRTEM, Tecnai G^2^F20 STEM, FEI Company, Hillsboro, OR, USA) with energy dispersive microanalysis system of X-ray (EDX) was used to investigate the interfacial structure of the cross-sectional samples and to determine the distribution of the fabricated diffusion samples in vertical section.

## Results and discussion

### Electrical properties

**Figure 1:**
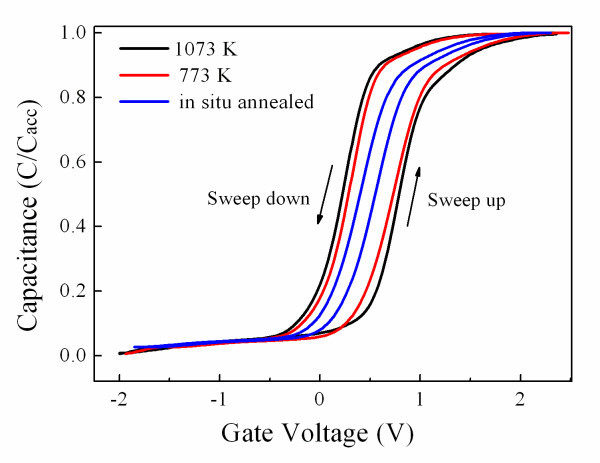
***C-V *curves of the TiAl**_**3**_**O**_***x***_**/Si capacitor**. Here, the relative capacitance *C*/*C*_acc _(practical capacitance value versus its accumulation capacitance) was chosen as *y*-axis in order to compare the different samples.

It is valuable to focus attention on those dielectric films with excellent electrical properties. Accordingly, the films with excellent properties are necessary and essential for the further microstructure investigation. The electrical hysteresis curves of the TiAl_3_O_*x *_films characterized by *C-V *were shown in Figure 1. The well behaved *C-V *curves were obtained by using serial mode of the MOS structure, where the hysteresis feature can be found after the gate voltage swept from inversion region to accumulation region and then swept back, regardless of post-annealing treatment upon the samples or not. It is observed that the sample without post-annealing treatment (*in situ *annealed) shows a very little hysteresis (about 0.15 V), while the films annealed at 773 and 1,073 K provide hysteresis windows of 0.57 and 0.6 V, respectively, which could be attributed to the formation of interfacial layer between the film and silicon substrate. The hysteresis windows of the *C-V *curves indicate that the flat-band voltage (*V*_FB_) responds to the charges injected into the films. The positive *V*_FB _shift indicates the existence of negative fixed charges in the film. These charges might be attributed to the oxygen vacancy. It is well known that the oxygen vacancy could be easily produced by the loss of oxygen at low oxygen partial pressure, and the oxygen vacancy will ionize and create the conducting electrons. This fact could bring on more defects staying in the TiAl_3_O_*x *_films and enhance the surface charge density of the film. Moreover, the films deposited by pulsed laser deposition have many defects inevitably, including the abovementioned oxygen vacancies. The existence of these defects makes the electron mobility of the films very active and convenient, and supplies the charges movement with more trenches, resulting in deteriorating the reliability of devices. By virtue of its capacitance-voltage curves, it is calculated that the oxide trapped charges density is about as much as 10^12^/cm^2^.

### Microstructural characteristics of the interface

**Figure 2:**
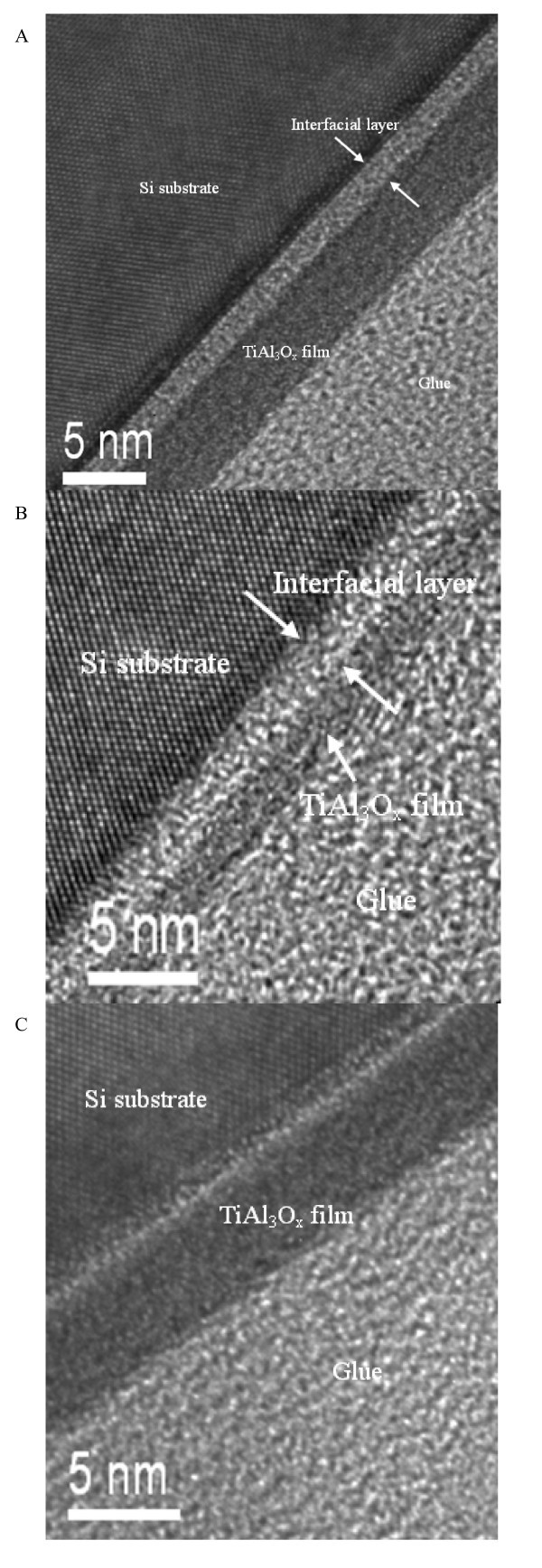
**HRTEM cross-section morphology of the TiAl**_**3**_**O**_***x***_**films**. Annealed at (**a**) 773 K and (**b**) 1,073 K, and (**c**) *in situ *annealed, respectively.

Actually, the speculation of changes on electrical properties derived from the referred three samples may achieve the further and straightforward confirmation by means of direct observation to inner structure of the films. Therefore, the cross-sectional images of the TiAl_3_O_*x *_film deposited on silicon substrate have been obtained by high-resolution transition electron microscopy as shown in Figure 2, respectively. For the samples with post-annealing treatment, the very thin interfacial layer about 0.6 nm (773 K) and 1.1 nm (1,073 K) can be observed between the film and the silicon substrate. Comparably, there is a very obscure interfacial layer for the sample without any thermal treatment. It is concluded that the thermal diffusion behavior has occurred at the region between the film and the silicon substrate after the thermal treatment, and therein, the interfacial layer thickness depends on the annealing temperature if the TiAl_3_O_*x *_film still keeps amorphous state [7].

### Characterization on thermal diffusion

From the high-resolution transition electron microscopy image, there is an obvious interfacial layer at film/silicon interface after the course of atoms diffusion upon thermal treatment. Similarly, a satisfied interfacial layer could be obtained, in the condition of enough film thickness and post-annealing time. Therefore, the scanning points are chosen from the bulk of the film, until the silicon substrate. Thus, the stable concentration could be regarded as the diffusion source along the direction perpendicular to the substrate.

As we all know, high-*k *materials on semiconductors are suitable for the fabrication of high speed, sub-45-nm complementary metal-oxide-semiconductor devices. However, the diffusion between high-*k *layer and semiconductor can cause an increase of leakage current and a decrease of the gate stack *k *value [8]. Thus, a convincing model solving the exact diffusion problem is helpful to understand the role of the post-annealing temperature upon the stack structure. The diffusion coupling model constructed with 2-μm-thick TiAl_3_O_*x *_films and silicon substrate could be regarded as the thin-film configuration model because the diffusion source is released at the surface and all the mass diffusion along the direction perpendicular to the substrate. The concentration field *C*(*x, t*) is considered as a function of time *t *and a single spatial variable *x*. So the Fick's second law under constant diffusivity for one-dimensional diffusion can be described as the form [9]:

(1) $$\frac{\partial C}{\partial t}=D\frac{\partial^{2}C}{\partial x^{2}}$$

In the experiment, an initial condition and one boundary condition are specified: (1) the initial state of the system is such that *C *= 0 for *x *> 0 when *t *= 0 (that is, *C*(x > 0,0) = 0), and (2) the left-hand boundary located at *x *= 0 is maintained at the fixed concentration *C*_0 _for all *t *> 0, that is, $\int_{0}^{\infty }C\left(x\right)d_{x}=C_{0}\left(t>0\right)$. At the same time, because the thickness of the film used in the experiment is about 2 μm, the effect of the Matano interface is ignored. So the diffusion solution of Equation 1 becomes:

(2) $$C\left(x,t\right)=\frac{C_{0}}{\sqrt{\pi Dt}}\exp\left(-\frac{x^{2}}{4Dt}\right)$$

where *D *is the diffusion coefficient. Therefore, by the linear relationship between the concentration and the diffusion distance at a certain time, the behavior of the thin-film couples may be analyzed in detail.

**Figure 3:**
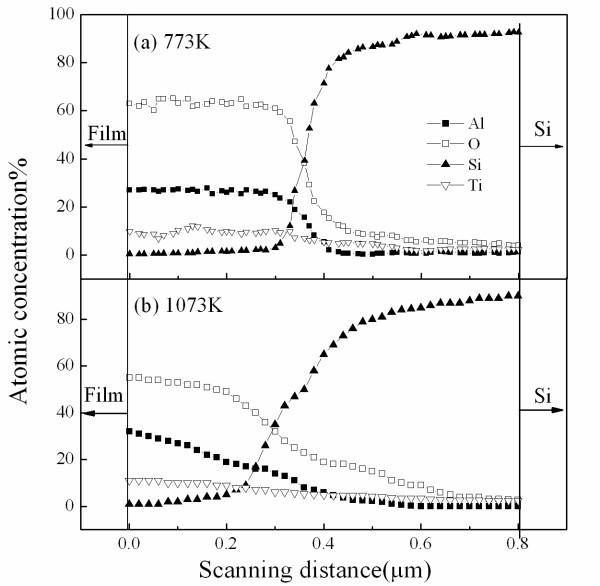
**Depth profiles of the TiAl**_**3**_**O**_***x***_**films**. Annealed at (**a**) 773 K and (**b**) 1,073 K, respectively.

The depth profiles of the TiAl_3_O_*x *_films have been recorded in Figure 3, derived from the EDX results. From the Figure 3a, the Si signal is close to zero in the bulk of the films and it sharply increases only close to the interface region due to the formation of the silicate layer. The concentrations of Ti and Al atoms are throughout the thickness of the films and decrease at the dielectric/substrate interface. However, in Figure 3b, the diffusion of the films annealed at 1,073 K is very obvious. The Ti and Al atoms have penetrated into Si substrate, and the Si atoms have entered into the body of the film as well.

**Figure 4:**
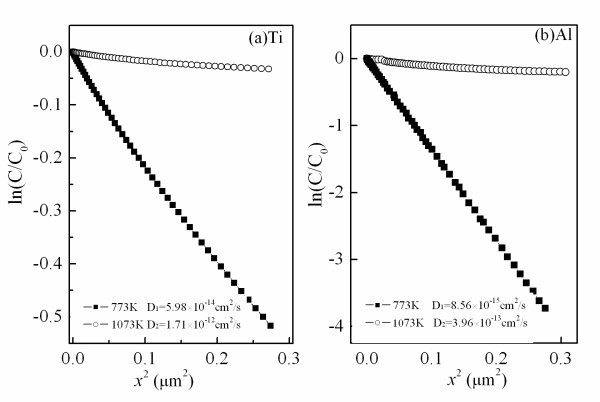
**Plot of the concentration versus the diffusion distance for the TiAl**_**3**_**O**_***x***_**films**. Annealed at 773 and 1,073 K, respectively: (**a**) Ti and (**b**) Al.

**Figure 5:**
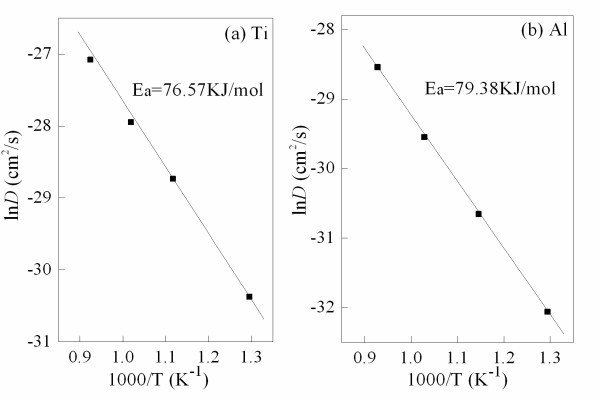
**Variation of the diffusion coefficients of TiAl**_**3**_**O**_***x***_**films as a function of the post-annealing temperature**. (**a**) Ti and (**b**) Al.

The concentration versus the distance for the Ti and Al atoms in silicon substrate of the same samples is shown in Figure 4. At the post-annealing temperature of 773 K, the diffusion coefficients of the Ti and Al atoms in silicon substrate are 5.98 × 10^-14 ^cm^2^/s and 8.56 × 10^-15 ^cm^2^/s, respectively. It indicates that these two kinds of atoms penetrate into silicon substrates about 0.29 and 0.11 μm per hour in the films annealed at 773 K along the direction perpendicular to the substrate. When the post-annealing temperature rises to 1,073 K, the diffusion coefficients of Ti and Al atoms increase to 1.71 × 10^-12 ^and 3.96 × 10^-13 ^cm^2^/s, and the diffusion distances of Ti and Al atoms approach to 1.56 and 0.75 μm, respectively. The values of the diffusion coefficients derived from the TiAl_3_O_*x *_films are larger than that derived from the crystalline solids [10]. It is presumed that the films deposited by the pulsed laser deposition method have many defects, such as oxygen vacancies. The existence of these defects makes the mobility of the atoms of the films very active and convenient. The variation of Ti and Al atoms in silicon substrates with the post-annealing temperature is shown in Figure 5. The diffusion coefficients steadily increase from 773 to 1,073 K, corresponding to the region of the kinetic control. An Arrhenius plot of each species gives the diffusion activation energy for Ti atoms in the TiAl_3_O_*x *_film of about 76.57 kJ/mol (0.79 eV/atom) and Al atoms of about 79.38 kJ/mol (0.82 eV/atom). The lower diffusion activation energy is related to the higher diffusion coefficient. This is ascribed to the fact that the diffusion in solid indeed takes place by atomic spring through the "saddle plane" energy barrier.

## Conclusions

In summary, with the *C*-*V *measurements, the electrical hysteresis of the TiAl_3_O_*x *_thin films has been revealed after high post-annealing temperature, which may be attributed to the oxide trapped charges density as much as 10^12^/cm^2^. The thermal diffusion behavior has occurred at the interfacial region after the thermal treatment, and therein, the interfacial layer thickness depends on the annealing temperature.

The thermal diffusion of metal atoms at TiAl_3_O_*x*_/Si interfacial region has been presented as well. The diffusion coefficient and the diffusion activation energy of the Ti and Al atoms derived from the films post-annealed at high temperature clearly disclose the diffusion behavior quantitatively.

## Competing interests

The authors declare that they have no competing interests.

## Authors' contributions

LS participated in the sequence experiments and measurements, and drafted the manuscript. ZL conceived of the study and designed some measurements. All authors read and approved the final manuscript
